# Phase sequences in balanced recurrent networks

**DOI:** 10.1186/1471-2202-14-S1-P207

**Published:** 2013-07-08

**Authors:** Nikolay Chenkov, Henning Sprekeler, Richard Kempter

**Affiliations:** 1 Institute for Theoretical Biology, Humboldt-Universität zu Berlin, Berlin, Germany; 2 Bernstein Center for Computational Neuroscience Berlin, Germany

## 

Electrophysiological recordings suggest that cortical circuits operate in a regime where the excitatory and inhibitory currents received by individual neurons are highly correlated in both time and stimulus selectivity. For such balanced input, neurons are activated by fluctuations in the input and tend to fire asynchronously and at irregular time intervals, a regime known as asynchronous irregular state.

**Figure 1:**
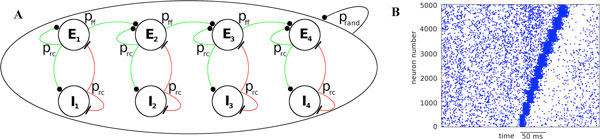
A. Schematic of network connectivity between excitatory (E) and inhibitory (I) groups; B. Raster plots of 5,000 excitatory neurons representing ten assemblies, each of size 500 neurons during a phase sequence (prc = 0.06, pff = 0.06, prand = 0.01).

However, transient synchronization and precise sequential firing has been observed during certain perceptual tasks or behavioral states. The neural mechanism behind these activity patterns has not yet been resolved. Here we address the problem by modeling a phase sequence embedded into a random recurrent network with sparse connectivity p_rand_. The phase sequence as originally proposed by Donald Hebb [1] is a series of activity of cell assemblies connected in a feed-forward fashion. In our model, neurons representing one assembly are connected with recurrent probability p_rc_>p_rand _while neurons in subsequent assemblies are connected with feed-forward probability p_ff_>p_rand_. Each assembly has a corresponding inhibitory subpopulation to which it is recurrently connected with probability p_rc _(see Figure 1A). Additionally, we apply an inhibitory plasticity rule that balances excitation and inhibition [2]. The role of such inhibition is twofold: it maintains asynchronous irregular firing of the excitatory population, and it enhances the response of an excitatory assembly through balanced amplification [3]. In the extreme case of no recurrent connectivity (p_rc _= 0), the network resembles a synfire chain: a feed-forward network with convergent-divergent connections between subsequent groups of neurons [4].

In contrast to synfire chain models, the balanced recurrent network dramatically reduces the connection probability p_ff _that is required for the propagation of activity (Figure 1B). Simulations reveal a range of parameters in which asynchronous irregular spiking coexists with reliable activation and propagation of synchronous waves.
